# Supplementary material for: The EDN2 rs110287192 gene polymorphism is associated with paratuberculosis susceptibility in multibreed cattle population
Source: PLoS One. 2020 Sep 3;15(9):e0238631. doi: 10.1371/journal.pone.0238631 (PMC7470282; doi:10.1371/journal.pone.0238631)
Supplement: S1 Table — F: forward; R: reverse; * assay IDs given by prob production company. (DOCX) [file pone.0238631.s001.docx]

**S1 Table. Primer and probes, used for genotyping of *EDN2* *rs109651404*, *rs110287192* and *rs136707411* SNPs.**

| **Gene** | **SNP** | **Primer (5'-3')** | **Reference** |
| --- | --- | --- | --- |
| ***EDN2*** | *rs109651404*  (ANKCAWX)* | F: 5'-GCAGGCAGTGACCTCAAAAG-3'  VIC-CTCCCAGTATGTCTTC  R: 5'-CCCAAAGCAGTGGGAGAGT-3'  FAM-CCCAGTGTGTCTTC | [20] |
| ***EDN2*** | *rs110287192*  (ANH6GCZ)* | F: 5'-TTTAGCAGCCTTTCCATACT-3'  VIC-CTTTTGGTGTACATAGCCACAA  R: 5'-CAGATCCAGGTAGATACAGAG-3'  FAM-CTTTTGGTGTACATATCCACAA | [20] |
| ***EDN2*** | *rs136707411*  (ANCFDT6)* | F: 5'-AGGCTCTGACTCTCCCAAGT-3'  VIC-ACCTCCCACACAGCAG  R: 5'-TGCTTTCCTGTCTGCCTACTG-3'  FAM-CCTCCCACGCAGCAG | - |

F: forward; R: reverse; * assay IDs given by probe production company.
