# Supplementary material for: The EDN2 rs110287192 gene polymorphism is associated with paratuberculosis susceptibility in multibreed cattle population
Source: PLoS One. 2020 Sep 3;15(9):e0238631. doi: 10.1371/journal.pone.0238631 (PMC7470282; doi:10.1371/journal.pone.0238631)
Supplement: Attachment — Submitted filename: PONE-D-20-15004 R1 Response to Reviewers.doc [file pone.0238631.s002.doc]

ONE-D-20-15004
The EDN2 rs110287192 Gene Polymorphism is Associated with Paratuberculosis Susceptibility in Multibreed Cattle Population
PLOS ONE

Dear Dr. Çınar,

Thank you for submitting your manuscript to PLOS ONE. After careful consideration, we feel that it has merit but does not fully meet PLOS ONE’s publication criteria as it currently stands. Therefore, we invite you to submit a revised version of the manuscript that addresses the points raised during the review process.


Please submit your revised manuscript by Aug 15 2020 11:59PM. If you will need more time than this to complete your revisions, please reply to this message or contact the journal office at plosone@plos.org. When you're ready to submit your revision, log on to https://www.editorialmanager.com/pone/ and select the 'Submissions Needing Revision' folder to locate your manuscript file.

Please include the following items when submitting your revised manuscript:

- A rebuttal letter that responds to each point raised by the academic editor and reviewer(s). You should upload this letter as a separate file labeled 'Response to Reviewers'.
- A marked-up copy of your manuscript that highlights changes made to the original version. You should upload this as a separate file labeled 'Revised Manuscript with Track Changes'.
- An unmarked version of your revised paper without tracked changes. You should upload this as a separate file labeled 'Manuscript'.

If you would like to make changes to your financial disclosure, please include your updated statement in your cover letter. Guidelines for resubmitting your figure files are available below the reviewer comments at the end of this letter.

If applicable, we recommend that you deposit your laboratory protocols in protocols.io to enhance the reproducibility of your results. Protocols.io assigns your protocol its own identifier (DOI) so that it can be cited independently in the future. For instructions see: [http://journals.plos.org/plosone/s/submission-guidelines#loc-laboratory-protocols](http://journals.plos.org/plosone/s/submission-guidelines#loc-laboratory-protocols)

We look forward to receiving your revised manuscript.

Kind regards,

Jasim Muhammad Uddin, DVM, PhD
Academic Editor
PLOS ONE

**Comment: Additional Editor Comments:**

**Comment 1**: There is a very strong link between Mycobacterium avium subspecies paratuberculosis in ruminant and Crohn's disease in humans but the cause of the later is not clear. However, have the authors found any Crohn's disease/like symptom in humans/farmers/animal handlers in the MAP-positive farm?

**Response 1**: There are strong clues that *Mycobacterium avium* subspecies *paratuberculosis* (MAP) the cause of a chronic intestinal disease in domestic and wild ruminants may cause of idiopathic inflammatory bowel disease (IIBD). Crohn’s disease is one of the IIBD form together with ulcerative colitis. However, in the present investigation, we did not follow up neither consumer’s health status nor farmers. To the best of our knowledge in Turkey, there is no study that investigate the prevalence of Crohn’s disease that caused by MAP. Nevertheless, in literature, experiments, were performed in other countries showed the MAP zoonotic transmission from domestic animals to humans.

Wynne JW, Bull TJ, Seemann T, et al. Exploring the zoonotic potential of Mycobacterium avium subspecies paratuberculosis through comparative genomics. PLoS One. 2011;6:e22171.

Pierce, E. S. (2018). Could Mycobacterium avium subspecies paratuberculosis cause Crohn’s disease, ulcerative colitis… and colorectal cancer? *Infectious Agents and Cancer*, *13*(1), 1-6.

In the manuscript, there is a sentence in the discussion “IBD is a chronic inflammatory disease of the gastrointestinal tract in humans that can be divided into those with Crohn’s disease, where disease may be present throughout the GI tract and those with Ulcerative Colitis, where disease is limited to the colon. There has been speculation that Crohn’s disease may be caused by MAP as well [34].”

34 Pierce, E. S. (2018). Could Mycobacterium avium subspecies paratuberculosis cause Crohn’s disease, ulcerative colitis… and colorectal cancer? *Infectious Agents and Cancer*, *13*(1), 1-6.

**Comment 2**: The public health importance would be good to discuss in the manuscript.
Though this study focused on association with SNPs, the details epidemiology of the disease in cattle population should be discussed. How animals were selected? Is there any prevalence study of this disease in the region? Is there any link with age, breed, nr in farm, husbandry practice, and human?

**Response 2**: Animals were selected according to clinical observations. Cattle, showed clinical signs of pTB as cachexia and diarrhea, were subjected to experiment. All, animals were in the same age; they were in 2-3 years old and exposed to the same environmental condition. The same region previously investigated by Gumussoy et al. (2015). There pTB prevalence was found 12.2% for Holstein and in our experiment, prevalence was identified as 11.7% which was cited in our previous article Cinar et al. 2018 and that was cited in the current manuscript as [21].

Gümüşsoy, K. S., Ica, T., Abay, S., Aydin, F., & Hizlisoy, H. (2015). Serological and molecular diagnosis of paratuberculosis in dairy cattle. *Turkish Journal of Veterinary and Animal Sciences*, *39*(2), 147-153.

Cinar MU, Hizlisoy H, Akyüz B, Arslan K, Aksel EG, Gümüşsoy KS. Polymorphisms in toll-like receptor (TLR) 1, 4, 9 and SLC11A1 genes and their association with paratuberculosis susceptibility in Holstein and indigenous crossbred cattle in Turkey. J Genet. 2018;97: 1147–1154.

Journal requirements:

When submitting your revision, we need you to address these additional requirements.

**Comment 1**: 1. Please ensure that your manuscript meets PLOS ONE's style requirements, including those for file naming. The PLOS ONE style templates can be found at

https://journals.plos.org/plosone/s/file?id=wjVg/PLOSOne_formatting_sample_main_body.pdf and

https://journals.plos.org/plosone/s/file?id=ba62/PLOSOne_formatting_sample_title_authors_affiliations.pdf

**Response 1**: Thanks for reminder, we have checked and modified accordingly.

**Comment 2**: 2. Thank you for stating the following in the Acknowledgments Section of your manuscript:

'Funding information: This research was financially supported by the Turkish Research Council (TUBITAK) grant number 218O128.

We note that you have provided funding information that is not currently declared in your Funding Statement. However, funding information should not appear in the Acknowledgments section or other areas of your manuscript. We will only publish funding information present in the Funding Statement section of the online submission form.

Please remove any funding-related text from the manuscript and let us know how you would like to update your Funding Statement. Currently, your Funding Statement reads as follows:

'The funders had no role in study design, data collection and analysis, decision to publish, or preparation of the manuscript.'

a. Please clarify the sources of funding (financial or material support) for your study. List the grants or organizations that supported your study, including funding received from your institution.
b. State what role the funders took in the study. If the funders had no role in your study, please state: “The funders had no role in study design, data collection and analysis, decision to publish, or preparation of the manuscript.”
c. If any authors received a salary from any of your funders, please state which authors and which funders.
d. If you did not receive any funding for this study, please state: “The authors received no specific funding for this work.”

**Response 2**: We have removed funding information from the acknowledgement and stated in the online Funding Statement section.

**Comment 3**: Please include your amended statements within your cover letter; we will change the online submission form on your behalf.

**Response 3**: We have given our amendment in the cover letter.

**Comment 4**: 3. Please include captions for your Supporting Information files at the end of your manuscript, and update any in-text citations to match accordingly. Please see our Supporting Information guidelines for more information: [http://journals.plos.org/plosone/s/supporting-information](about:blank)

**Response 4**: Revised accordingly.

**Comment 5**: 4. Your ethics statement must appear in the Methods section of your manuscript. If your ethics statement is written in any section besides the Methods, please move it to the Methods section and delete it from any other section. Please also ensure that your ethics statement is included in your manuscript, as the ethics section of your online submission will not be published alongside your manuscript.

**Response 5**: Thanks for reminder. I have deleted the “Compliance with ethical standards:” section in the revised version of manuscript.

**Review Comments to the Author**

Please use the space provided to explain your answers to the questions above. You may also include additional comments for the author, including concerns about dual publication, research ethics, or publication ethics. (Please upload your review as an attachment if it exceeds 20,000 characters)

**Reviewer #1**

**Comment 1**: Reviewer #1: Line 86-90: Do you have any data for your country (Turkey) to show how impact of this disease in your country?

**Response 1**: There are few literatures about the local (province level) prevalence of paratuberculosis in Turkey, done by using different detection methods such as serology and PCR. But those experiments have subjected limited number of animals and farms. Additionally, no comprehensive sampling and analyzing method was applied in national level, therefore, no economic analysis had been generated in which deals with the loss which is caused by paratuberculosis in Turkey.

**Comment 2**: Line 139: Total animal is 818 animals but in this line number you have written 68 infected and 753 healthy animals so 68+753 is not 818.

**Response 2**: Sorry for inconvenience. There was a typing error. The number of healthy animals were corrected as 750 in the revised manuscript.

**Comment 3**: Result Part: You have 3 breeds and why not give the result for the association genotyping result individual breed. Individual association for breeding might be give strong results that SNP will affect on these 3 breeds or not. This is better to analysis SNP in each breed.

**Response 3**: Thank for the comment. There were two reasons not to apply single breed analysis instead of multi-breed analysis. First, the number of cases were lower in native-crossbreds compared to Holsteins. There were 7 pTB cases and 4 pTB cases were detected in East Anatolian Red crosbred and Anatolian Black crosbred, respectively. On the other hand, 57 pTB cases were identified in the Holsteins. Hence, we thought, since our aim was to identify association of the mutations with the pTB status, we used multi-breed approach. Second, researcher stated the advantages of using multiple breedsfor association analysis in literature and applied this method in many animal species (Ramayo-Caldas et al. 2016, Bianchi et al. 2015, van den Berg, Boichard, Lund, 2016).Karlsson et al. (2007) stated that using multiple breeds that share the same phenotype, may decrease the noise, and help to identify selected loci accurately.

Karlsson, E., Baranowska, I., Wade, C. et al. Efficient mapping of mendelian traits in dogs through genome-wide association. Nat Genet 39, 1321–1328 (2007).

Ramayo-Caldas, Y., Renand, G., Ballester, M., Saintilan, R., & Rocha, D. (2016). Multi-breed and multi-trait co-association analysis of meat tenderness and other meat quality traits in three French beef cattle breeds. *Genetics Selection Evolution*, *48*(1), 37.

Bianchi, M., Dahlgren, S., Massey, J., Dietschi, E., Kierczak, M., Lund-Ziener, M., & Ollier, W. E. (2015). A multi-breed genome-wide association analysis for canine hypothyroidism identifies a shared major risk locus on CFA12. PLoS One, 10(8), e0134720.

van den Berg, I., Boichard, D., & Lund, M. S. (2016). Comparing power and precision of within-breed and multibreed genome-wide association studies of production traits using whole-genome sequence data for 5 French and Danish dairy cattle breeds. Journal of Dairy Science, 99(11), 8932-8945.

**Reviewer #2**

**Comment 1**: The authors mentioned the aims of this study to analyse a potential association between EDN2 gene with pTB. To support more justification EDN2 as a candidate gene, could more explain about the position of EDN2 in chromosome is related with pTB and why the mutation that author chose synonymous mutation

**Response 1**: In livestock, *EDN2* has been investigated for its corpus luteum formation in cattle. However, no research was devoted for the association of bovine *EDN2* with immune traits. EDN2 mutations, selected for the current work taken from the previous studies. Settles *et al*. [18] and Neibergs *et al*. [19] reported *EDN2* as a strong functional and positional candidate gene for pTB susceptibility in Holstein cattle according to GWA study. Three *EDN2* synonymous mutations, named *rs110287192, rs109651404* and *rs109490418,* were patented for being associated with pTB susceptibility in Holstein breed cattle [20].

18.	Settles M, Zanella R, McKay SD, Schnabel RD, Taylor JF, Whitlock R, et al. A whole genome association analysis identifies loci associated with *Mycobacterium avium* subsp. *paratuberculosis* infection status in US holstein cattle. Anim Genet. 2009;40: 655–662. doi:10.1111/j.1365-2052.2009.01896.x

19.	Neibergs HL, Settles ML, Whitlock RH, Taylor JF. GSEA-SNP identifies genes associated with Johne’s disease in cattle. Mamm Genome. 2010;21: 419–425. doi:10.1007/s00335-010-9278-2

20.	Neibergs HL, Ricardo Z, Taylor JF, Wang Z, Scraggs E, White SN, et al. Compositions and methods for diagnosis of genetic susceptibility, resistance, or tolerance to infection by mycobacteria and bovine paratuberculosis using promoter variants of EDN2. Google patents [Preprint]. 2014 [cited 2020 April 17]. Available: https://patents.google.com/patent/US20140283151

**Response 2**: Although EDN2 has been investigated for its corpus luteum formation in cattle

**Comment 2**: - The sample were collected from three regions. How about the environment condition, are they in the same condition. Please explain more clearly

**Response 2**: All animals were subjected to same environmental condition. Samples were taken in summer and they were kept in the same farm that they were fed with the same ratio. “Animals included in the present study were housed in similar environmental conditions” statement has already been mentioned in the sample collection section.

**Comment 2**: - For genotyping used custom Taqman SNP genotyping assay. It was not really clear mentioned in the methodology why used this method?

**Response 2**: The mutations, were genotyped in the present work derived from a previous GWAS study. TaqMan genotyping method could be one of the most cost and time efficient method for these kinds of mutations identified by GWAS. TaqMan system is one of the earliest methods for SNP genotyping, based on fluorescently-tagged, allele-specific probes detected using real-time polymerase chain reaction (PCR)-based assays. Since mutations did not genotyped before elsewhere, no literature mentioned about RFLP enzyme for DNA digestion. Therefore, we had to designed custom TaqMan assays for the respective mutations.

The sentence was added in the revised manuscript: “Since current SNPs have not been genotyped by using any other method, the custom TaqMan chemistry was selected as cost and time effective genotyping method.”

Osaki, R., Imaeda, H., Ban, H., Aomatsu, T., Bamba, S., Tsujikawa, T., & Andoh, A. (2011). Accuracy of genotyping using the TaqMan PCR assay for single nucleotide polymorphisms responsible for thiopurine sensitivity in Japanese patients with inflammatory bowel disease. *Experimental and therapeutic medicine*, *2*(5), 783-786.

**Comment 3**: - In statistical part using proc logistic. Are this statistical model is common to used by previous study and authors were not put also the mathematical model for these analysis. Any specific reason why using Proc Logistic because normally to perform association using GLM model

**Response 3**: Binary logistic regression is useful where the dependent variable is dichotomous (e.g., healthy/sick, live/die, graduate/dropout, vote for A or B). Proc Logistic is very similar to Proc GLM, although it has a binary outcome variable rather than an interval outcome.If the outcome is ordinal, Proc Logistic can also be used, but with a complementary log-log link function instead of the more standard log function. Logistic regression also defines odds ratios for the input variables. There are similar articles analyze the same type of data by using Proc Logistic.

Vázquez P, Ruiz-Larrañaga O, Garrido JM, et al. Genetic association analysis of paratuberculosis forms in holstein-friesian cattle. *Vet Med Int*. 2014;2014:321327. doi:10.1155/2014/321327

Ruiz-Larrañaga O, Garrido JM, Manzano C, et al. Identification of single nucleotide polymorphisms in the bovine solute carrier family 11 member 1 (SLC11A1) gene and their association with infection by Mycobacterium avium subspecies paratuberculosis. *J Dairy Sci*. 2010;93(4):1713-1721. doi:10.3168/jds.2009-2438

Bhaladhare A, Sharma D, Kumar A, et al. Single nucleotide polymorphisms in toll-like receptor genes and case-control association studies with bovine tuberculosis. *Vet World*. 2016;9(5):458-464. doi:10.14202/vetworld.2016.458-464

Mohd Baqir, Saket Bhushan, Amit Kumar, Arvind Sonawane, Ranvir Singh, Anuj Chauhan, Ramji Yadav, Om Prakash, Renjith R, Aashish Baladhare & Deepak Sharma (2016) Association of polymorphisms in SLC11A1 gene with bovine tuberculosis trait among Indian cattle, Journal of Applied Animal Research, 44:1, 380-383, doi: 10.1080/09712119.2015.1091333

**Comment 4**: - Paten number should be written in the methodology

**Response 4**: The sentence was modified in the revised manuscript.

From:intergenic variant and *rs109490418*–g.104706758G>A in 3′ UTR variant mutations were patented by [20]

To:intergenic variant and *rs109490418*–g.104706758G>A in 3′ UTR variant mutations were patented by Neibergs *et al*. patent# US20140283151 [20]

**Comment 5**: - In Table 1, the number of animal between SNP that authors examined were different. Why the number of animal were different for example the first, second and third SNP were 796; 769 and 751 respectively

**Response 5**: Thanks for this point. The reason why animal number showing variability was the missing genotypes. Due to limited budget, we had no chance to repeat animals. However, animal numbers per genotype is still over 200, which is recommended for reliable association analysis.

**Comment 6**: - By perform association study, is it sufficient to determine by choosing rs110287192-GG genotype may decrease the risk pTB in cattle without support functional study such as expression study mRNA and protein. Could authors extend to see how the expression between healthy and infected. We suggest validating also on functional study using significant SNP rs110287192 by comparing infected and healthy animals.

**Response 6**: The current experiment was aimed to identify the favorable genotype for pTB resistancy. Reviewer is right to ask whether EDN2 was the correct candidate gene for pTB susceptibility. Authors are planning for to continue the functional genomic analysis such as RNA-Seq and Western Blot analysis for EDN2 gene. However, this needs animal should be bought from farmers after validated to be pTB. Further financial applications are warranted to perform functional genomics experiments in order to investigate pTB susceptibility.
