# Supplementary material for: The EDN2 rs110287192 gene polymorphism is associated with paratuberculosis susceptibility in multibreed cattle population
Source: PLoS One. 2020 Sep 3;15(9):e0238631. doi: 10.1371/journal.pone.0238631 (PMC7470282; doi:10.1371/journal.pone.0238631)
Supplement: Attachment — Submitted filename: PONE-D-20-15004 R2 Response to Reviewers.doc [file pone.0238631.s004.doc]

PONE-D-20-15004R1
The EDN2 rs110287192 Gene Polymorphism is Associated with Paratuberculosis Susceptibility in Multibreed Cattle Population

**Additional Editor Comments** (if provided):

**Comment 1**: No more comments from me but please address the queries from Dr Gunawan.
**Response 1**: Thanks editor for improving the quality of manuscript.

**Reviewer Comments:**
**Reviewer #1:**

**Comment 1**: The author was response from the reviewers clearly and corrected according to the suggestion as well. The manuscript is currently in scientific standard for publication in this journal. However, the next scientific report in deeply still needed. Hope the author using the scientific to solve this problem in livestock production.

**Response 1**: Thanks reviewer for improving the quality of manuscript. We are continuing to work in pTB, we are going to inform Ministry of Agriculture in Turkey in terms of our results. They may evaluate results and may suggest to use in selection programs.

**Reviewer #2:**

**Comment 1**: The authors have revised their manuscript and taken some, but not all of my earlier comments into account.

**Response 1**: We tried to answer all points. Sorry for inconvenience, we answer all points in this version.

**Comment 2**: 1. It is still unclear what the the EDN2 position or is located in the BTA (Bos Taurus) chromosom. Please meantion it in which chromosome?. If the authors mentioned are taken from GWAS study explain litle bit information in the manuscript to support these gene is important related to pTB.

**Response 2**: Chromosome location of three EDN2 mutations were given and details of previous GWAS study was mentioned in the manuscript. The sentence was modified as:

In addition, Settles *et al*. [18] and Neibergs *et al*. [19] reported *EDN2* as a strong functional and positional candidate gene for pTB susceptibility in Holstein cattle according to GWAS study. *EDN2* locus on bovine chromosome 3 was identified with genome-wide significant level of association to the presence of MAP in tissue and both tissue and feces respectively [18]. Three *EDN2* synonymous mutations on bovine chromosome 3 (BTA3), named *rs110287192, rs109651404* and *rs109490418* were patented for being associated with pTB susceptibility in Holstein breed cattle [20].

**Comment 3**: 2. Authors have explained that the animal which used in this study were in the same environment condition. The sample were collected in summer time based on reviewed response. Could authors added in the manuscript including also the age of cattle to emphesized that only effect of genetic EDN2 that would be obsreved.

**Response 3**: Age of cattle was mentioned in the sample collection sub-section in line 132 in the revised manuscript.

**Comment 4**: 3. In line 224-225, authors observed suggestive association between the EDN2
rs136707411 and increased pTB risk. Its better to put the level of p-value which is categorized as suggestive association forisntance (P<0.10) etc

**Response 4**: p value was added in the revised version (L228).

**Comment 5**: 4. Line 251-252: No genotype of EDN2 rs109651404 were found to be significant
252 associated with pTB (all p > 0.05). This sentence could be merge with previous paragraph because only consist few sentences.

**Response 5**: The sentence was merged with the previous paragraph as asuggested.

**Comment 6**: 5 Line 308-311, Further analyses through expanded sampling of other cattle breeds are recommended to better understand the role genomic selection could play in reducing the susceptibility to pTB in cattle. Based on previous comment please add not only the large number of cattle which should be added, but also it is needed functional study of EDN2 at the level of mRNA and protein expression. Authors could seen paper related below:
Gunawan, A., K. Kaewmala, M.J. Uddin, M. U. Cinar, D. Tesfaye, C. Phatsara, E. Tholen, C. Looft, and K. Schellander. 2011. Association study and expression analysis of porcine ESR1 as a candidate gene for boar fertility and sperm quality. Anim. Reprod. Sci. 128: 11-21.
Gunawan, A., K. Kaewmala, M.J. Uddin, M. U. Cinar, D. Tesfaye, C. Phatsara, E. Tholen, C. Looft, and K. Schellander. 2011. Investigation on association and expression of ESR2 as a candidate gene for boar sperm quality and fertility. Domest. Reprod. Anim. doi:10.1111/j.1439-0531.2011.01968.x

**Response 6**: There is no experimental infection unit in Turkey suitable for bovine experiments. However, we are planning to collect slaughterhouse tissue and organ samples from pTB positive and negative animals too. Thanks for sample articles and suggestion. We have revised the sentence accordingly.

Further analyses that are combining genotyping and holistic gene expression methods through expanded sampling of other cattle breeds together with blood mRNA and serum protein samples are recommended to better understand the role genomic selection could play in reducing the susceptibility to pTB in cattle.
