# Supplementary material for: The EDN2 rs110287192 gene polymorphism is associated with paratuberculosis susceptibility in multibreed cattle population
Source: PLoS One. 2020 Sep 3;15(9):e0238631. doi: 10.1371/journal.pone.0238631 (PMC7470282; doi:10.1371/journal.pone.0238631)
Supplement: Attachment — Submitted filename: Reviewer Comments ASG 11.08.2020.docx [file pone.0238631.s003.docx]

The authors have revised their manuscript and taken some, but not all of my earlier comments into account.

1. It is still unclear what the the EDN2 position or is located in the BTA (Bos Taurus) chromosome. Please mention it in which chromosome?. If the authors mentioned are taken from GWAS study explain little bit information in the manuscript to support these gene is important related to pTB.

2. Authors have explained that the animal which used in this study were in the same environment condition. The sample were collected in summer time based on reviewed response. Could authors added in the manuscript including also the age of cattle to emphasized that only effect of genetic EDN2 that would be observed.

3. In line 224-225, authors observed suggestive association between the EDN2

rs136707411 and increased pTB risk. Its better to put the level of p-value which is categorized as suggestive association foresentence (P<0.10) etc

4. Line 251-252: No genotype of EDN2 rs109651404 were found to be significant

252 associated with pTB (all p > 0.05). This sentence could be merge with previous paragraph because only consist few sentences.

5 Line 308-311, Further analyses through expanded sampling of other cattle breeds are recommended to better understand the role genomic selection could play in reducing the susceptibility to pTB in cattle. Based on previous comment please add not only the large number of cattle which should be added, but also it is needed functional study of EDN2 at the level of mRNA and protein expression. Authors could seen paper related below:

Gunawan, A., K. Kaewmala, M.J. Uddin, M. U. Cinar, D. Tesfaye, C. Phatsara, E. Tholen, C. Looft, and K. Schellander. 2011. Association study and expression analysis of porcine ESR1 as a candidate gene for boar fertility and sperm quality. Anim. Reprod. Sci. 128: 11-21.

Gunawan, A., K. Kaewmala, M.J. Uddin, M. U. Cinar, D. Tesfaye, C. Phatsara, E. Tholen, C. Looft, and K. Schellander. 2011. Investigation on association and expression of ESR2 as a candidate gene for boar sperm quality and fertility. Domest. Reprod. Anim. doi:10.1111/j.1439-0531.2011.01968.x
